# Temporal trend analysis of avoidable mortality in Taiwan, 1971-2008: overall progress, with areas for further medical or public health investment

**DOI:** 10.1186/1471-2458-13-551

**Published:** 2013-06-06

**Authors:** Brian K Chen, Chun-Yuh Yang

**Affiliations:** 1 Department of Health Services Policy and Management, Arnold School of Public Health, University of South Carolina, 800 Sumter Street #116, Columbia, SC 29208, USA; 2 Department of Public Health, College of Health Sciences, Kaohsiung Medical University, 100 Shih-Chuan 1st Road, Kaohsiung 807, Taiwan

**Keywords:** Avoidable mortality, Standard estimated years of life lost, Taiwan, Lung cancer, Breast cancer

## Abstract

**Background:**

Avoidable mortality (AM), or “unnecessary untimely death,” is considered an indicator of health care quality. We investigated trends in the age-standardized mortality rates (ASMRs) and associated standard expected years of life lost (SEYLL) for deaths amenable to medical care or public health measures in Taiwan from 1971-2008, with an emphasis on identifying areas where additional medical or public health investment may help reduce the burden of AM.

**Methods:**

Taiwan’s ASMRs per 100,000 for AM and other causes of death were calculated using data from the National Death Certificate Registry in five-year bins from 1971 to 2008. SEYLL rates per 100,000 were calculated annually from 1971 to 2008 using the same data source.

**Results:**

ASMR for almost all AM and other causes of death declined dramatically from 1971 to 2008 except for lung cancer (16.6% and 7.4% increase among men and women, respectively) and breast cancer (109.8% increase among women). In the same period, SEYLL due to lung cancer increased from 269.2 to 555.7 for men and 249.7 to 342.5 for women. For women, SEYLL due to breast cancer increased from 263.5 in 1971 to 659.3 in 2008. There were gender-specific differences in the reduction (or increase) in AM rates, with women showing larger rates of reduction or smaller rates of increase. Among men, AM fell by 65.9% from 1971-1975 to 2006-2008, and deaths from other causes increased by 15.6%. Among women, AM and deaths from other causes fell by 80.8% and 59.8% respectively. SEYLL decreased, respectively among males and females, from 23,147.3 and 24,081.1 in 1971 to 11,261.8 and 5,929.6 in 2008.

**Conclusion:**

From 1971 to 2008, Taiwan experienced a dramatic reduction in most AM and corresponding SEYLL except for lung cancer (for both males and females) and breast cancer (for females). Additional effort should be devoted to public health measures to combat the rising prevalence of smoking in Taiwan, which may be responsible for the increasing AM from lung cancer. If AM in breast cancer continues unabated in the future, greater policy emphasis on the early detection and treatment of breast cancer may also be warranted.

## Background

Avoidable mortality (AM), or “unnecessary untimely deaths” that could have been prevented with appropriate medical care or public health measures, is considered an indicator of health care quality [1]. Temporal trend studies documented that AM generally fell faster than other causes of death during the last few decades in the West [2-5]. Mackenbach and colleagues, in particular, demonstrated that reductions in AM often followed introduction of effective treatments for the associated medical conditions [3]. As a whole, the literature credits the increases in health services effectiveness for much of the decline in avoidable deaths in Western countries [3,5,6]. Nevertheless, few studies have investigated the trend in AM in Asian countries, which together represent over 60% of world population [7].

The primary aims of this study were twofold. First, we documented the temporal trends in AM and associated changes in standard expected years of life lost (SEYLL) in Taiwan from 1971 to 2008. Second, we identified specific disease categories where additional medical or public health investment may be warranted given their unsatisfactory AM trends.

Studies linking reductions in AM with access to care or socioeconomic status have been widely conducted since the 1970s in North America and Europe [5,6,8-20]. Only a few studies, however, investigated the trend in AM in Asian populations. A Korean study revealed that AM generally declined from 1983 to 2004, with the exception of ischemic heart disease and several forms of malignant neoplasms (lung, breast, and cervix) [21]. Another compared the age-standardized avoidable mortality rates and proportions in four cities around the world, and found that Hong Kong had the highest rates in AM from cerebrovascular disease [22]. In Taiwan, Lee and colleagues [23] argued that the introduction of National Health Insurance (NHI) accelerated the decline of AM, particularly among the young and the elderly, populations unlikely to have been insured under the previous employment-based health insurance regime. No study, however, has documented the temporal trends in AM by individual disease categories in Taiwan. Our study fills this void in the literature in order to identify areas for further medical or public health investment to reduce the burden of AM in Taiwan.

## Methods

We obtained mortality data from the Taiwan National Death Certification Registry between 1971 and 2008. The population-based registry contains data elements including gender, year of birth, and the date and cause of death for all residents of Taiwan. According to Taiwan law, a death certificate must be issued and registered within 30 days after the death of a resident. Trained medical registrars review and code all death certificates in the central office of the National Death Certification Registry. As a result, cause-of-death coding is considered very accurate in Taiwan [24]. To categorize deaths into “avoidable deaths” and “deaths due to other causes,” we followed the definition established by the Concerted Action of the European Community on Avoidable Mortality (CAEC) [25,26]. The CAEC built on the work pioneered by Charlton and colleagues [2], which identified deaths that were thought to be amenable to public health policy programs as well as a wide array of health services, such as primary care and hospital services. As in James and colleagues [27], we considered all AM, including those amenable to medical care, those amenable to public health, and those amenable to both.

Such “untimely unnecessary deaths” include, for example, deaths from ischemic heart disease (IHD). These deaths can be prevented or delayed though public health interventions such as smoking cessation programs [28,29] or through medical interventions such as prescription drugs [30] or surgical procedures [31]. Untimely deaths from several forms of malignant neoplasms, including breast, cervical, and uterine cancers can be avoided through screening and treated with radiation therapy, surgery or chemotherapy [32-34]. The two principal categories of AM primarily amenable to public health programs are lung cancer and accidental injuries, the former through smoking cessation initiatives [35] and the latter through safety legislation [36]. The list of these amenable deaths is reproduced in Table 1.

**Table 1:** Selected avoidable deaths

**Cause of death**	**Intervention**	**ICD9 code**	**Age-groups**
***Deaths amenable to both medical care and public health***		001-999	0-64
Ischemic heart disease	Public health	410-414, 429.2	35-64
	Smoking cessation, lifestyle modification		
	Medical care		
	Pharmacotherapy, angioplasty, surgery		
***Deaths amenable to medical care***			
Tuberculosis	Immunization, contact tracing, pharmacotherapy	010-018, 137	5-64
Malignant neoplasm of the breast [breast cancer]	Screening, surgery, radiation therapy, chemotherapy	174	25-64
Malignant neoplasm of the cervix uteri [cervical cancer]	Screening, surgery, radiation therapy, chemotherapy	180	15-64
Malignant neoplasm of the uterus [uterine cancer]	Screening, surgery, radiation therapy, chemotherapy	179, 182	15-64
Hodgkin's disease	Chemotherapy, radiation therapy,	201	15-64
Hypertension and cerebrovascular disease	Pharmacotherapy, carotid endarterectomy	401-405, 430-438	35-64
Asthma	Pharmacotherapy	493	5-44
Gastric and duodenal ulcer [ulcers]	Pharmacotherapy, surgery	531-534	25-64
Appendicitis	Surgery	540-543	5-64
Hernia	Surgery	550-553	5-64
Cholelithiasis, cholecystitis and cholangitis [Gallbladder diseases]	Pharmacotherapy, surgery	575-476	5-64
Complications of pregnancy [Maternal mortality]	Pharmacotherapy, surgery	630-676	0-64
Perinatal conditions	Screening, pharmacotherapy, surgery	760-779	> 28 weeks, gestation < 1 week and stillbirths
***Deaths amenable to public health***			
Malignant neoplasm of the trachea, bronchus and lung [Lung cancer]	Smoking cessation, lifestyle modification	162	35-64
Accidents/Poisonings/Violence [Injuries]	Legislation, safety equipment	800-999	0-64

Adapted from the European Community Working Group on Health Services and Avoidable Deaths, 1997 and reproduced from James et al [27].

Because our data span nearly four decades, we restricted our analyses to AM occurring at age 65 or under despite the considerable advances in life expectancy in Taiwan. Deaths in Taiwan between 1971 and 2008 not due to AM were classified as “deaths from other causes.” The numbers of AM and other deaths sum to the number of all-cause deaths. Several variations exist on the definition of AM, including a more recent 2004 update [37]. However, given the length of the study period, we selected the 1997 CAEC list of AM as the basis for our analysis.

Trends in age-standardized all-cause mortalities, AM, and deaths due to other causes were separated into five-year bins beginning in 1971, except the last bin (2006-2008). Separate analyses were conducted for males and females because some medical conditions (such as breast cancer or cervical cancer) are gender-specific, and others may have differential outcomes based on an individual’s gender (such as cerebrovascular diseases [38] or traffic-related deaths [39]). Age-standardized mortality rates (ASMRs) per 100,000 individuals were calculated using the direct method [40], as follows:

$$\text{ASMR}=\frac{\sum W_{i}\times A_{i}}{\sum W_{i}}$$

where W_i_ is the population in the *i*th age class of the reference population (the world population in 2000) and A_i_ is the age-specific mortality rate in the *i*th age class in Taiwan.

Standard expected years of life lost (SEYLL) were calculated annually using the following formula:

$$\mathrm{SEYLL}=\sum_{i\leq 1}^{70-74}E_{i}\times \frac{d_{i}}{p_{i}}\times \frac{P_{\mathrm{Ri}}}{N_{R}}$$

where E_i_ is the 2008 life expectancy in Taiwan for the mid-age of age group *i*, d_i_ is the number of deaths from the cause in the age group *i*, p_i_ is the number of people in the examined population age group i, P_Ri_ is the number of people in the 2000 world reference population age group *i*, and N_R_ is the number of people in the reference population under 75 years of age.

We present all ASMR and SEYLL values, and compare gender differences without confidence intervals or statistical tests because our data include the entire population of all deaths in Taiwan from the National Death Certification Registry.

## Results

**Figure 1:**
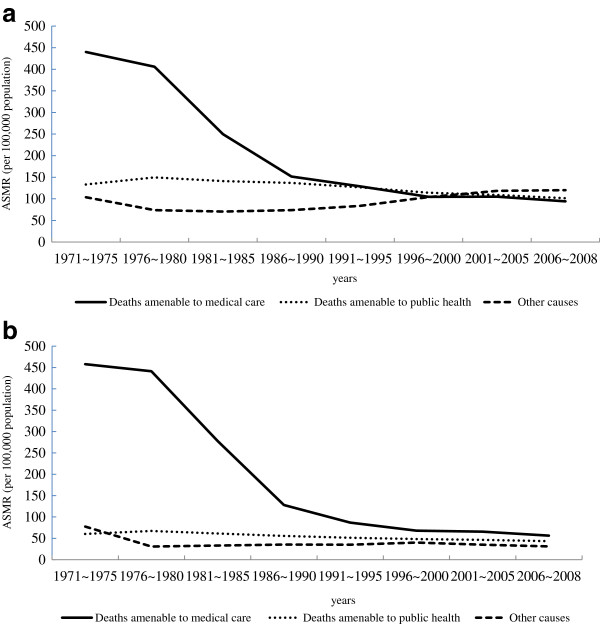
Deaths amenable to public health, deaths amenable to medical care, and unavoidable deaths from 1971 to 2008 (a: males, b: females).

**Figure 2:**
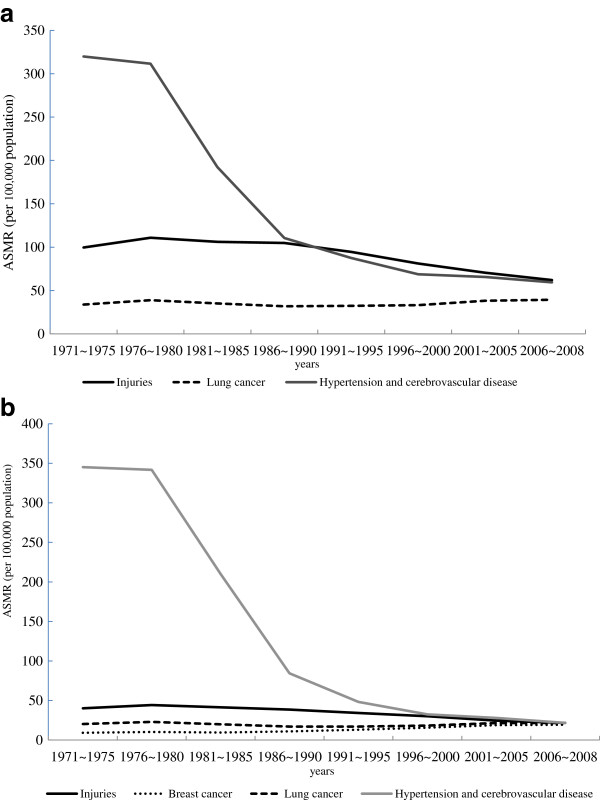
Deaths due to lung cancer, hypertension/cerebrovascular disease, and injuries, 1971-2008 (a: males, b: females).

The temporal changes in AM amenable to medical care, public health, and other causes of death are presented graphically in Figure 1a and b for men and women, respectively. In Figure 2a and b, AM trends are shown for four diseases that either experienced the greatest reductions in AM (hypertension/cerebrovascular disease and injuries) or increased during the study period (lung cancer for both men and women, and breast cancer for women). Equivalent figures expressed in SEYLL rates are presented in Additional files 1 and 2.

Tables 2 and 3 show the same data in tabular form, including the trends in all-cause mortalities, AM, and deaths due to other causes in five-year increments (except the last three years) from 1971 to 2008, for males and females respectively. Annual SEYLL rates from 1971 to 2008 are presented in the Additional file 3 for males and Additional file 4 for females. The ASMRs for all but two amenable deaths followed a consistent trend downward during the 38-year study period. The only infectious disease on the list of AM, tuberculosis, experienced dramatic reductions from 50.3 (7.4% of all deaths for males) and 25.4 (4.3% for females) per 100,000 in 1971-1975 to 1.4 (0.3%) and 0.3 (0.2%) in 2006-2008 respectively. In fact, among men, tuberculosis demonstrated one of the largest reductions (100%) in ASMR during the study period. SEYLL due to tuberculosis dropped from 1,478.3 in 1971 to 40.0 in 2008. Among women, the reduction in AM due to tuberculosis was also dramatic (98.8%), with the corresponding SEYLL falling from 1,020.8 in 1971 to 12.5 in 2008.

**Table 2:** All-cause mortality under 65 years and “avoidable” mortality in Taiwan from 1971 to 2008, males

**Cause**	**1971~1975**		**1976~1980**		**1981~1985**		**1986~1990**		**1991~1995**		**1996~2000**		**2001~2005**		**2006~2008**		**% change**
	**ASMR**	**%**	**ASMR**	**%**	**ASMR**	**%**	**ASMR**	**%**	**ASMR**	**%**	**ASMR**	**%**	**ASMR**	**%**	**ASMR**	**%**	
All causes	677.4	100	629.5	100	461.7	100	362.2	100	339.8	100	323.4	100	332.4	100	315.6	100	53.4
Injuries	99.6	14.7	110.9	17.6	106.1	23	104.9	29	94.5	27.8	81.2	25.1	70.6	21.2	62.1	19.7	37.7
Ischemic heart disease	36.7	5.4	43.8	7	33.6	7.3	29.3	8.1	32.5	9.6	28.7	8.9	33.6	10.1	30.6	9.7	16.6
Lung cancer	33.8	5	38.9	6.2	35.1	7.6	31.9	8.8	32.3	9.5	33.2	10.3	38.3	11.5	39.4	12.5	-16.6
Hypertension and cerebrovascular disease	319.9	47.2	311.6	49.5	192.3	41.7	110.6	30.5	87.5	25.8	68.7	21.2	65.7	19.8	59.4	18.8	81.4
Ulcers	27.3	4	16	2.5	7.9	1.7	3.5	1	3.1	0.9	2.8	0.9	2.2	0.7	1.8	0.6	93.4
Hodgkin's disease	0.3	0	0.3	0	0.2	0	0.1	0	0.1	0	0.1	0	0.1	0	0.1	0	66.7
Asthma	3.9	0.6	1.1	0.2	0.7	0.2	0.6	0.2	0.6	0.2	0.5	0.2	0.5	0.2	0.5	0.2	87.2
Gallbladder disease	1.1	0.2	1	0.2	0.6	0.1	0.3	0.1	0.3	0.1	0.3	0.1	0.3	0.1	0.3	0.1	72.7
Tuberculosis	50.3	7.4	31.6	5	14.3	3.1	7.1	2	5	1.5	3.7	1.1	2.6	0.8	1.4	0.4	97.2
Hernia	0.3	0	0.2	0	0.1	0	0	0	0	0	0	0	0	0	0	0	100
Appendicitis	0.4	0.1	0.3	0	0.2	0	0.1	0	0	0	0	0	0	0	0	0	100
“Avoidable” causes	573.6	84.7	555.7	88.3	391.1	84.7	288.4	79.6	255.9	75.3	219.2	67.8	213.9	64.4	195.6	62	65.9
Deaths amenable to medical care	440.2	65.0	405.9	64.5	249.9	54.1	151.6	41.9	129.1	38.0	104.8	32.4	105	31.6	94.1	29.8	78.6
Deaths amenable to public health	133.4	19.7	149.8	23.8	141.2	30.6	136.8	37.8	126.8	37.3	114.4	35.4	108.9	32.8	101.5	32.2	23.9
Other causes	103.8	15.3	73.8	11.7	70.6	15.3	73.8	20.4	83.9	24.7	104.2	32.2	118.5	35.6	120	38	-15.6

**Table 3:** All-cause mortality under 65 years and “avoidable” mortality in Taiwan from 1971 to 2008, females

**Cause**	**1971~1975**		**1976~1980**		**1981~1985**		**1986~1990**		**1991~1995**		**1996~2000**		**2001~2005**		**2006~2008**		**% change**
	**ASMR**	**%**	**ASMR**	**%**	**ASMR**	**%**	**ASMR**	**%**	**ASMR**	**%**	**ASMR**	**%**	**ASMR**	**%**	**ASMR**	**%**	
All causes	596	100	539.2	100	372	100	219.1	100	173.2	100	156.3	100	146.7	100	130.9	100	78
Injuries	40.1	6.7	44.3	8.2	41.5	11.2	38.6	17.6	34.3	19.8	30.1	19.3	24.6	16.8	21.7	16.6	45.9
Breast cancer	9.2	1.5	10.3	1.9	9.6	2.6	10.9	5	13.2	7.6	15.6	10	18.9	12.9	19.3	14.7	-109.8
Lung cancer	20.3	3.4	23	4.3	19.9	5.3	17.1	7.8	17	9.8	18.4	11.8	21.6	14.7	21.8	16.7	-7.4
Ischemic heart disease	31.3	5.3	40.1	7.4	29.1	7.8	17	7.8	12.5	7.2	9.1	5.8	9.2	6.3	7.8	6	75.1
Hypertension and cerebrovascular disease	345.1	57.9	341.8	63.4	210	56.5	84.4	38.5	48.3	27.9	32.5	20.8	28	19.1	21.7	16.6	93.7
Cervical cancer	9.5	1.6	10.2	1.9	8.7	2.3	7.7	3.5	8.1	4.7	7.1	4.5	6.1	4.2	4.4	3.4	53.7
Hodgkin's disease	0.2	0	0.1	0	0.1	0	0.1	0	0	0	0	0	0	0	0	0	100
Ulcers	15.7	2.6	10.1	1.9	5.6	1.5	1.4	0.6	0.9	0.5	0.7	0.4	0.7	0.5	0.5	0.4	96.8
Asthma	5.4	0.9	1.4	0.3	0.7	0.2	0.5	0.2	0.4	0.2	0.4	0.3	0.3	0.2	0.2	0.2	96.3
Gallbladder disease	1.7	0.3	1.6	0.3	0.7	0.2	0.3	0.1	0.2	0.1	0.1	0.1	0.2	0.1	0.2	0.2	88.2
Maternal mortality	2.1	0.4	1.3	0.2	0.7	0.2	0.3	0.1	0.2	0.1	0.2	0.1	0.1	0.1	0.1	0.1	95.2
Tuberculosis	25.4	4.3	14	2.6	6.1	1.6	2.4	1.1	1.4	0.8	1.1	0.7	0.6	0.4	0.3	0.2	98.8
Hernia	0.1	0	0.1	0	0.1	0	0	0	0	0	0	0	0	0	0	0	100
Uterine cancer	11.6	1.9	9.9	1.8	6	1.6	2.9	1.3	1.6	0.9	1.1	0.7	1.5	1	1.7	1.3	85.3
Appendicitis	0.6	0.1	0.4	0.1	0.2	0.1	0.1	0	0	0	0	0	0	0	0	0	100
“Avoidable” causes	518.3	87	508.6	94.3	339	91.1	183.7	83.8	138.1	79.7	116.4	74.5	111.8	76.2	99.7	76.2	80.8
Deaths amenable to medical care	457.9	76.8	441.3	81.8	277.6	74.6	128	58.4	86.8	50.1	67.9	43.4	65.6	44.7	56.2	42.9	87.7
Deaths amenable to public health	60.4	10.1	67.3	12.5	61.4	16.5	55.7	25.4	51.3	29.6	48.5	31.0	46.2	31.5	43.5	33.2	28.0
Other causes	77.7	13	30.6	5.7	33	8.9	35.4	16.2	35.1	20.3	39.9	25.5	34.9	23.8	31.2	23.8	59.8

### AM with the greatest reductions

In terms of the absolute number of ASMR reductions, deaths from hypertension and cerebrovascular diseases dropped from 319.9 per 100,000 (47.2% of all deaths) in 1971-1975 to 59.4 per 100,000 (18.8% of all deaths) in 2006-2008, a fall of 81.4% among men. SEYLL due to hypertension and cerebrovascular disease among men dropped from 7,586.2 in 1971 to 1,920.8 in 2008. Among women, AM from hypertension and cerebrovascular diseases also witnessed significant reductions – from 345.1 per 100,000 (57.9% of all deaths) to 21.7 per 100,000 (16.6% of all deaths), or a decline of 93.7%. For women, SEYLL for hypertension-related diseases also fell noticeably from 9,774.5 in 1971 to 966.3 in 2008. The ASMR for ischemic heart disease also (IHD) also fell for both men and women, but there were noticeable disparities between the sexes. The IHD ASMR dropped from 36.7 per 100,000 (5.4% of all deaths) to 30.6 per 100,000 (9.7% of all deaths) for men, a reduction of 16.6%. Among men, although the ASMR for IHD fell during this period, concomitant with a rise in the proportion of deaths due to IHD, SEYLL due to IHD increased from 767.4 in 1971 to 989.8 in 2008. For women, however, the change was far more dramatic. From 31.3 per 100,000 (5.3% of all deaths), the IHD ASMR plunged to 7.8 per 100,000 (6.0% of all deaths), a decline of nearly 75.1%. Among women, IHD SEYLL declined from 841.2 in 1971 to 379.9 in 2008. For both sexes, even as the ASMRs declined, the proportion of deaths due to ischemic heart disease increased.

Injuries are another area where there were large declines in ASMRs and significant differences between the sexes. In almost every single period, the ASMR due to injuries for men was more than twice that for women. In 1971-1975, 99.6 per 100,000 men died from injuries (14.7% of all deaths), and 40.1 per 100,000 women (6.7% of all deaths) lost their lives from injuries. The reduction in injury-related ASMRs over the nearly four-decade study period was 37.7% for men, and 45.9% per women. SEYLL rates for both men and women declined, respectively, from 3,933.7 and 1,823.6 in 1971 to 2,194.0 and 901.8 in 2008.

Other AM that experienced large reductions in percentage terms include ulcers (93.4% reduction for men, 96.8% for women), hernia (100% reduction for men, 100% for women), appendicitis (100% reduction for men, 100% for women), asthma (87.2% reduction for men, 96.3% for women), Hodgkin’s disease (66.7% reduction for men, 100% for women), and gallbladder disease (72.2% reduction for men, 88.2% for women). AM with reductions of 100% are a result of rounding, signifying that mortality from these causes is virtually nil at the population (aged 65 and under) level by 2006-2008.

### AM that experienced increases

The only two categories of AM that countered the continual decline in ASMRs throughout the period were deaths due to lung cancer (for both men and women) and to breast cancer (for women only). Lung cancer ASMRs grew from 33.8 per 100,000 (5.0% of all deaths) among men in 1971-1975 to 39.4 per 100,000 (12.5% of all deaths) in 2006-2008, an increase of 16.6%. SEYLL from lung cancer doubled, from 269.2 in 1971 to 555.7 in 2008 among men. For women, the increase was smaller, but still in the high single digits at 7.4%, from 20.3 per 100,000 (3.4% of all deaths) to 21.8 per 100,000 (16.7% of all deaths). SEYLL likewise increased, from 249.7 in 1971 to 342.5 in 2008 among women. The growth in ASMR for breast cancer among women, in percentage terms, was dramatic. From 9.2 per 100,000 (1.5% of all deaths) in 1971, the number jumped 109.8% to 19.3 per 100,000 (or 14.7% of all deaths). SEYLL as a result of breast cancer increased 2.5-fold, from 263.5 in 1971 to 659.3 in 2008.

### Other gender-specific AM and gender disparities in AM

Among types of mortality specific to women, almost all ASMRs fell from 1971 to 2008. Cervical cancer declined from 9.5 per 100,000 (1.6% of all deaths) in 1971-1975 to 4.4 per 100,000 (3.4% of all deaths) for a reduction of 53.7% by 2006-2008. Maternal mortality dropped as well, by nearly 95.2%, from 2.1 per 100,000 (0.4% of all deaths) to 0.1 per 100,000 (or 0.1% of all deaths). Uterine cancer fell 85.3%, from 11.6 per 100,000 (1.9% of all deaths) to 1.7 per 100,000 (1.3% of all deaths). AM from some diseases (such as cervical cancer) experienced drops in ASMRs but grew as a percentage of all deaths because deaths from other diseases fell more quickly.

Gender disparities exist in the relative reductions both in AM and in other deaths. In the study period, AM reductions were greater among women (80.8%) than among men (65.9%) in the aggregate, but also for both AM amenable to medical care (87.7% for women vs. 78.6% reduction for men) and AM amenable to public health interventions (28% for women vs. 23.9% reduction for men). While deaths from other causes fell by 59.8% for women, among men such deaths increased by 15.6%. Aggregate SEYLL from AM dropped both for men and women, respectively from 14,878.4 and 15,609.9 in 1971 to 5,772.1 and 3,531.1 in 2008, again showing greater improvements for women than for men.

## Discussion

Our study findings show an encouraging downward trend in most types of AM and their associated SEYLL rates from 1971 to 2008 in Taiwan. As in many rapidly developing countries, the mortality burden shifted from infectious to chronic diseases. The spectacular reduction in deaths due to tuberculosis followed both the introduction of an effective treatment for the disease (isoniazid) [41] and a mass Bacille Calmette-Guerin vaccination program [42]. Among chronic illnesses, the most impressive reductions in ASMRs occurred for deaths due to hypertension and cerebrovascular diseases between 1976-1980 and 1986-1990. During this period, hypertension and cerebrovascular ASMR for males fell by 81.4%, from 319.9 per 100,000 in 1976-1980 to 59.4 per 100,000 in 1986-1990. It dropped by almost 93.7% for women, from 345.1 per 100,000 to 21.7 per 100,000. Between 1976 and 1990, SEYLL rates from hypertension and cerebrovascular diseases declined by 47.6% among men and by 61.3% among women.

Prior research suggests that the dramatic reduction in hypertension-related deaths during this period was a result of a successful anti-hypertension campaign [43]. Deaths from stroke, a cerebrovascular disease, fell rapidly from 1982 because of the reduction in the case-fatality rate of hemorrhagic stroke (HS), which was three to four times higher than the case-fatality rate of cerebral infarction (CI) during this period [43]. Despite a lower prevalence of HS (35.5% of acute strokes in Taiwan) relative to CI, it constituted 60.1% of all fatal strokes [43]. Hypertension was the most significant risk factor for virtually all episodes of acute stroke, and was most frequently associated with patients with HS. On the other hand, CI was more frequent among patients with comorbidities such as diabetes mellitus, cardiac disease and hyperlipidemia. The combination of rapidly decreasing HS case-fatality rates (through the anti-hypertension campaign), and a relative increase in CI to HS since 1982 together appear to explain much of the reduction in AM due to hypertension and cerebrovascular diseases in the 1980s.

To verify whether the impressive reductions in AM from hypertension and cerebrovascular diseases from 1971 to 1990 resulted from changes in coding within the National Death Certificate Registry, we searched the literature on the quality of death certificate coding in Taiwan. A study of 5,621 random sample (5%) of total deaths in 1994 showed a high level of agreement between the original coders and the reviewers, with 14.85% and 29.20% false positive for deaths due to cerebrovascular diseases and hypertension-related diseases, and 0.85% and 0.4% false negative [24]. The relatively high false positive figures signify that as of 1994, reductions in hypertension- and cerebrovascular AM may well have been underestimated. The literature search yielded no major systematic changes in coding procedure in Taiwan until the late 1990s, when the Department of Health initiated an evaluation of the quality of cause-of-death reporting by certifying physicians and the quality of the coding procedure [44]. Without systematic coding procedure changes for these two disease categories between 1971 and 1990, random variations in coding procedures alone are unlikely to result in the impressive fall in AM in this period.

Another important cause of deaths that witnessed significant declines since 1971 is mortality from accidental injuries. This trend is particularly prominent among men. Male ASMRs for injuries hovered around 100-110 per 100,000 for much of the late 1970s to the 1990s, until they fell to 94.5 in the 1991-1995. A similar pattern is seen among women. Until 1986-1990, deaths from accidental injuries remained around 40 per 100,000. Then, in 1991-1995, the figure fell to 34.3 per 100,000. SEYLL associated with injuries varied by approximately 100 from year to year, until it dropped by 345.69 in 1997 for men. For women, the changes were less dramatic, but SEYLL variations hovered between the single digits and 100 in the 1990s until a drop of 132 in 1996 and 110 in 1997. During this period, large numbers of the Taiwanese relied on motorcycles as their primary means of transportation. Traffic injuries also represented the primary cause of death for accidental injuries [39]. The sharp drop in SEYLL in 1997, in particular for males, coincided with implementation of a mandatory motorcycle helmet law, which may have been a contributing factor to the reduction in AM from injuries during the study period [36]. A study of the 1997 law showed that motorcycle fatalities decreased by 14% post-implementation of the safety legislation [45].

### Areas for further medical and public health investment to reduce AM

#### Potential reasons for AM increases from lung and breast cancers

We now return to the two causes of death that rose from 1971 to 2008 – lung cancer (for both men and women) and breast cancer (for women). Globally, cancer of the lung is overwhelmingly caused by tobacco smoking [46-51]. Once diagnosed, it has an overall five-year survival rate of only 16.3% according to U.S. estimates [52], and 21.3% and 23.6% for men and women respectively in Taiwan [53]. The prevalence of cigarette smoking in Taiwan rose dramatically since the Second World War [54]. By 2001, the estimated prevalence of adult smokers was 46.8% for males and 4.3% and females [55]. The male-to-female ratio of smokers was markedly lower for adolescents at 14.3% to 4.0% [55], signifying that differences between male and female lung cancer mortality rates may further narrow in the future. Because no effective medical treatment exists for lung cancer once diagnosed, public health measures, particular tobacco control, may be the best policy to counter the rise in lung cancer-related AM in Taiwan [15,27,35,56,57].

As opposed to lung cancer, breast cancer is much more amenable to medical care through screening, surgery, radiation therapy and chemotherapy. If detected sufficiently early (stage 0), breast cancer has a 5-year survival rate of 93%, which decreases to 88%, 81%, 74%, 67%, 41%, 49%, and 15% as the disease progresses to stages I, IIA, IIB, IIIA, IIIB, IIIC, and IV [58]. Among Taiwanese women, the overall 5-year survival rate is 80.4%, and 95.7%, 93.9%, 88.5%, 65%, and 18.5% respectively for stages 0, I, II, III and IV [59].

The increase in AM from breast cancer *may* represent a missed opportunity in Taiwan. The literature generally substantiates better survival rates for breast cancer among Asian women than among Caucasian women [60,61]. Taiwan is also considered a low-incidence area for breast cancer [62]. Estimated age-adjusted incidence was 15-20 per 100,000 in Taiwan in 1993-1994 [62], relative to 60-90 per 100,000 in the United Kingdom and the United States in 1985 [57]. Moreover, mortality rates from breast cancer remain lower in Taiwan than in the United States. In 2006-2008, the ASMR for breast cancer was 19.3 per 100,000 in Taiwan (See Table 3), but 22.4 and 31.6 per 100,000 among white and black women respectively in the United States based on data from 2005-2009 [63].

Yet despite lower incidence and mortality rates in Taiwan, ASMRs for breast cancer grew by 23.7% between in 1996 and 2008 even as these rates have declined steadily by 2.0% per year among women in the United States (2.1% among white women and 1.4% per year among black women) between 1999 and 2008 [64]. A combination of lifestyle changes, advances in therapy (adjuvant systemic therapy), earlier detection of palpable tumors, as well as mammography screening have been credited for the decline in breast cancer ASMRs in the United States and other developed countries [65]. It is possible that the higher rates of breast cancer ASMR in Taiwan may be due to increased testing and more accurate cause-of-death coding in recent years. Because the benefits of interventions to detect and treat breast cancer in the early stages may not be appreciated for many years, close monitoring of temporal trends in AM for this disease should continue in Taiwan. More aggressive strategies to detect and treat breast cancer in its earlier stages, particularly among women with the highest risk, may be warranted if reversals in the increases in breast cancer deaths do not occur.

### Gender disparities in ASMR

In virtually all non-gender-specific causes of death, there is a clear pattern of gender disparities in ASMRs at baseline and in the reduction in or increase in ASMRs over time. In the 38-year period, all-cause mortality rates decreased by 53.4%, avoidable causes, by 62.0%, and other causes increased by 15.6% among men. During the same period, these three figures fell respectively by 78.0%, 80.8%, and 59.8% for women. For the four leading non-gender-specific causes of avoidable deaths (injuries, hypertension/cerebrovascular disease, lung cancer and ischemic heart disease), ASMRs were (a) already lower among women at baseline and decreased more among women over the years (injuries), (b) roughly equal at baseline, with women exhibiting a greater reduction in death rates (cerebrovascular and heart disease), or (c) increased at a slower rate than men (lung cancer) over the 38-year period. These results are broadly consistent with findings of higher male than female mortality rates in developed countries [66].

With respect to the gender differences in ASMRs for cerebrovascular diseases, the result is particularly interesting in light of numerous reports of more severe functional outcomes and incidence of stroke among women [38,67,68]. Part of the reason may be that women tend to suffer from strokes and other cerebrovascular disease at an older age than men [67], and we specifically limited our analysis of ASMRs to deaths of those aged 65 and under. Future research should investigate whether this gender disparity in cerebrovascular mortality holds for a more elderly portion of the Taiwanese population.

## Conclusion

We found impressive reductions in AM and SEYLL in Taiwan from 1971 to 2008, particularly for deaths due to injuries and hypertension- and cerebrovascular-related diseases. Effective traffic safety legislation in 1997 and a successful anti-hypertension campaign in the 1980s may be partially credited for these successes. Nevertheless, the study also identified two notable exceptions to the falling AM trends in Taiwan – lung cancer among both men and women, and breast cancer among women. The increase in lung cancer mortality rates is likely due to the rising prevalence of smokers in Taiwan, and to the lack of effective medical treatment for lung cancer once detected. The increase in breast cancer ASMRs, however, may represent a missed opportunity for Taiwan to reduce mortality from this particular form of cancer among women, particularly if such trends do not reverse in the near future.

## Limitations and future research

When interpreting the results of this study, a few factors must be considered. First, despite Taiwan’s household registration system and an associated vital statistics data repository (death certification registry) that are considered excellent, coding errors do occur [24,69]. However, to alter the basic conclusions of this study, there would have had to be systematic undercoding in the latter years of AM that experienced reductions in ASMRs, and systematic overcoding of AM that witnessed increases. Second, despite Taiwan’s implementation of National Health Insurance (NHI) in 1996, a visual inspection of the AM trend lines suggests that NHI did not noticeably reduce amenable deaths at the aggregate population level. Future research should consider the potential differential impact of insurance coverage expansion on vulnerable populations such as the indigent, the elderly, and ethnic minorities.

Given the increase in life expectancy in Taiwan, future research should also consider raising the ASMRs to age 75. Likewise, to identify potential differential impacts of increased access to care, disease-specific studies should be conducted by dividing the study sample into groups that were already covered under the old patchwork of insurance plans and those who were newly insured in 1996. Geographic variations, as well as socioeconomic differences in AM ASMRs should also be studied, since any potential positive impact of the NHI may be masked in an aggregate analysis such as ours. Finally, the underlying mechanisms differentiating the gender differences in ASMR trends should also be studied, as they may offer important policy insight to improve the public’s health.

## Abbreviations

AM: Avoidable mortality; ASMR: Age-standardized mortality rate; CAEC: Concerted action of the European community on avoidable mortality; NHI: National Health Insurance; SEYLL: Standard estimated years of life lost.

## Competing interests

The authors declare that they have no competing interests.

## Authors’ contributions

Dr. Chen reviewed the empirical results of the avoidable mortality, devised the conceptual framework for the research article, and composed the manuscript. Dr. Yang collected the data, performed the empirical analysis, and provided comments for revisions of the manuscript. Both authors read and approved the final manuscript.

## Authors’ informations

Dr. Brian Chen

JD (Stanford Law School)

PhD (University of California at Berkeley)

Assistant Professor, University of South Carolina

As an applied economist, Dr. Chen’s research focuses on the impact of incentives in health care organizations on provider and patient behavior. For his dissertation, Chen empirically examined how vertical integration and prohibition against self-referrals affected physician prescribing behavior. His job market paper was selected for presentation at the American Law and Economics Association’s Annual Meeting, the Academy of Management, the Canadian Law and Economics Association, the Conference on Empirical Legal Studies, and the First Annual Conference on Empirical Health Law and Policy at Georgetown Law Center in 2009. The paper was also nominated for best paper based on a dissertation at the Academy of Management.

Dr. Chen jointed the University of South Carolina not only with a multidisciplinary law and economics background, but also with an international perspective from having lived and worked in Taiwan, Japan, and France. He has a deep-seated interest in exploring the impact of laws and regulations on the organization, provision, and productivity of health care in the United States and abroad.

Dr. Chun-Yuh Yang

Ph.D., Epidemiology and Public Health, School of Medicine, Yale University, USA. M. P.H., Public Health, School of Medicine, National Taiwan University, Taiwan. B.Sc., Public Health, National Taiwan University School of Medicine, Taiwan

Dr. Yang devotes his energies to the environmental epidemiologic studies last decade. The main interests of his researches are the health outcomes resulting from air pollution and various contaminants in drinking water.

Dr. Yang has published approximately 100 SCI papers for which he is the first or corresponding author as yet. In addition, he served as an editorial board member for several SCI and domestic journals and is a regular reviewer for many others.

## Pre-publication history

The pre-publication history for this paper can be accessed here:

http://www.biomedcentral.com/1471-2458/13/551/prepub

## Supplementary Material

Additional file 1 **Stanford expected years of life lost by type of mortality (a: males, b: females).** National Death Certificate Registry, 1971-2008. Data include cause of death of all mortality in Taiwan from 1971 to 2008. Click here for file

Additional file 2 **Standard expected years of life lost due to injuries, lung cancer, and hypertension (a: males, b: females).** National Death Certificate Registry, 1971-2008. Data include cause of death of all mortality in Taiwan from 1971 to 2008. Click here for file

Additional file 3 **Avoidable mortality (SEYLL rate per 100000 people), males.** National Death Certificate Registry, 1971-2008. Data include cause of death of all mortality in Taiwan from 1971 to 2008. Click here for file

Additional file 4 **Avoidable mortality (SEYLL rate per 100000 people), females.** National Death Certificate Registry, 1971-2008. Data include cause of death of all mortality in Taiwan from 1971 to 2008. Click here for file
