# Supplementary figures and images for: Temporal trend analysis of avoidable mortality in Taiwan, 1971-2008: overall progress, with areas for further medical or public health investment
Source: BMC Public Health. 2013 Jun 6;13:551. doi: 10.1186/1471-2458-13-551 (PMC3744173; doi:10.1186/1471-2458-13-551)

**(a)**


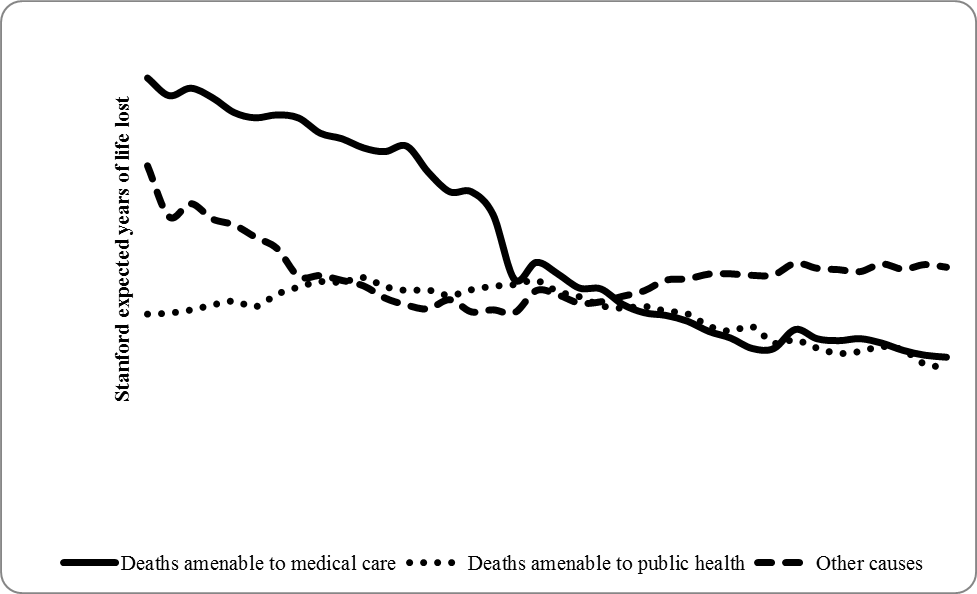


**(b)**


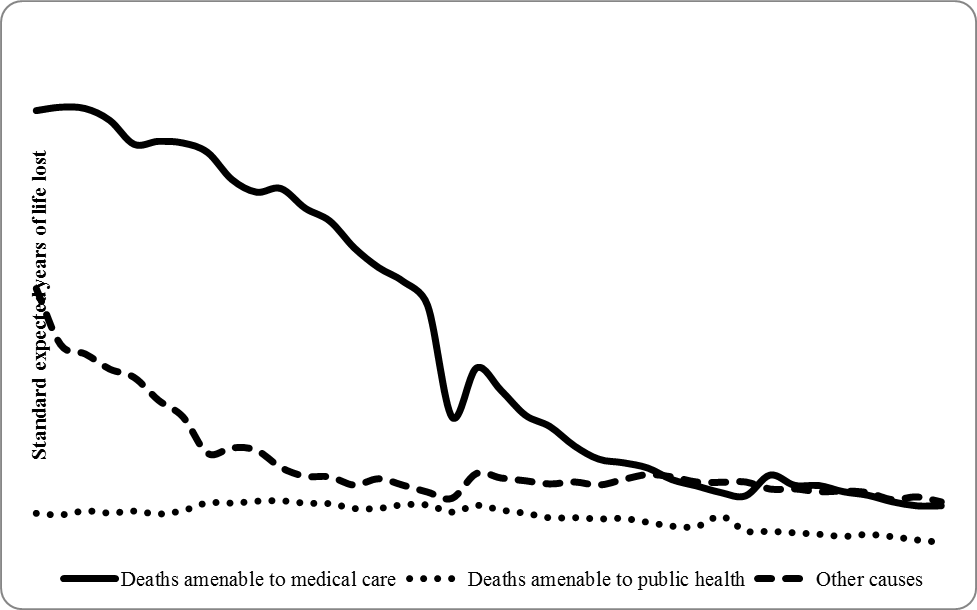

Supplement: Additional file 1 — Stanford expected years of life lost by type of mortality (a: males, b: females). National Death Certificate Registry, 1971-2008. Data include cause of death of all mortality in Taiwan from 1971 to 2008. [file 1471-2458-13-551-S1.doc]

**(a)**


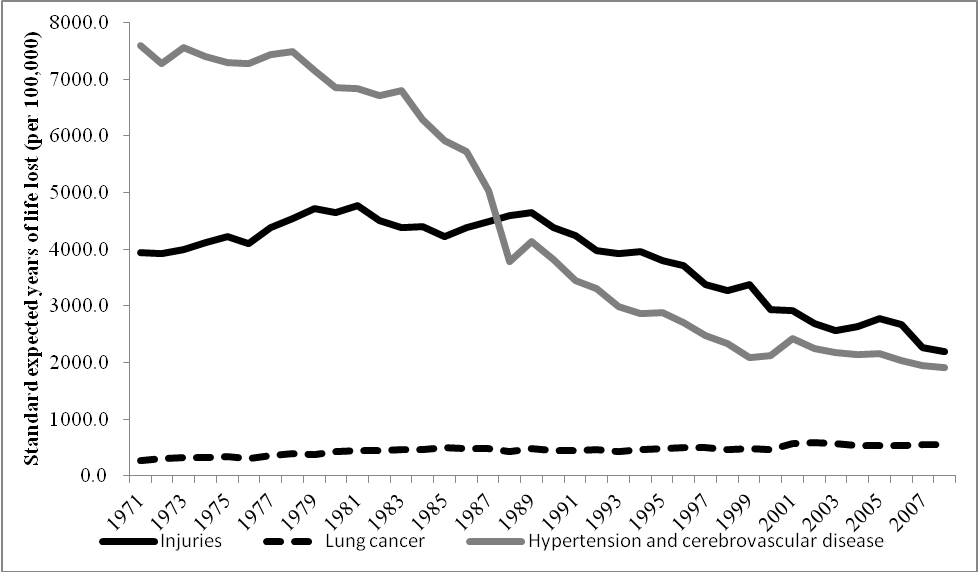


**(b)**


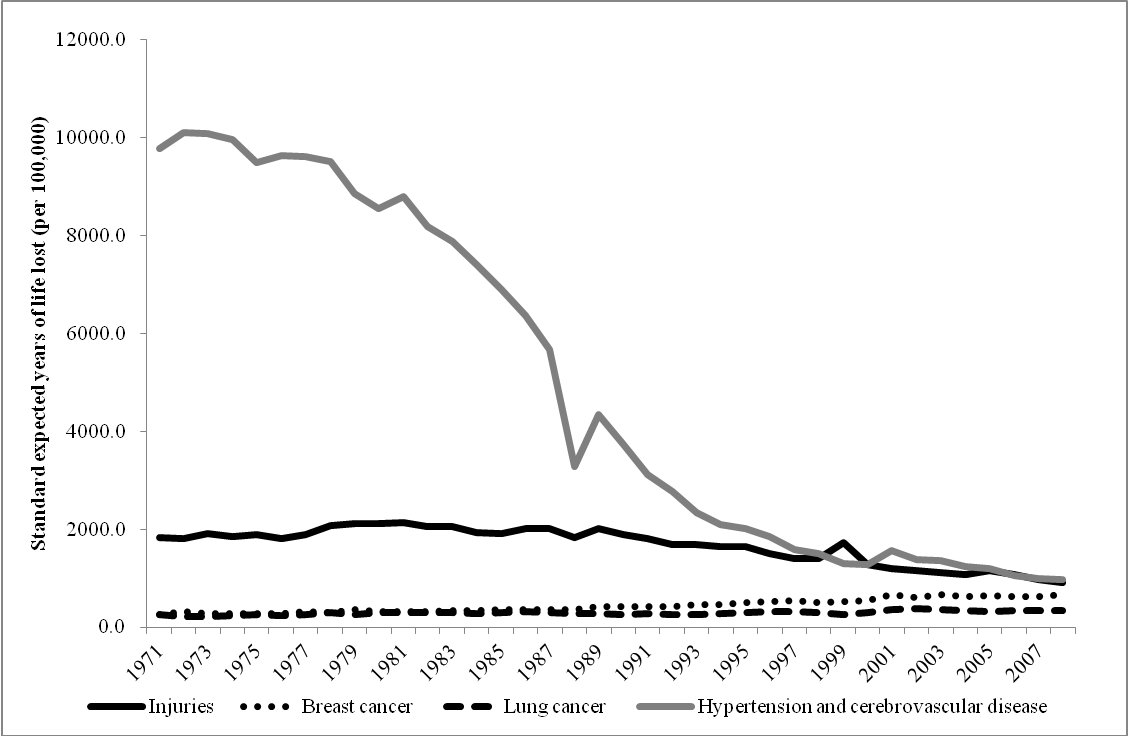

Supplement: Additional file 2 — Standard expected years of life lost due to injuries, lung cancer, and hypertension (a: males, b: females). National Death Certificate Registry, 1971-2008. Data include cause of death of all mortality in Taiwan from 1971 to 2008. [file 1471-2458-13-551-S2.doc]
