# Supplementary material for: Temporal trend analysis of avoidable mortality in Taiwan, 1971-2008: overall progress, with areas for further medical or public health investment
Source: BMC Public Health. 2013 Jun 6;13:551. doi: 10.1186/1471-2458-13-551 (PMC3744173; doi:10.1186/1471-2458-13-551)
Supplement: Additional file 3 — Avoidable mortality (SEYLL rate per 100000 people), males. National Death Certificate Registry, 1971-2008. Data include cause of death of all mortality in Taiwan from 1971 to 2008. [file 1471-2458-13-551-S3.pdf]

Additional file 3: Avoidable mortality (SEYLL rate per 100000 people), males.

| Cause | 1971 | 1972 | 1973 | 1974 | 1975 | 1976 | 1977 | 1978 |
|---|---|---|---|---|---|---|---|---|
| | Year | | | | | | | |
| All causes | 23147.3 | 21287.9 | 21946.9 | 21401.3 | 20945.0 | 20297.7 | 20415.5 | 19758.8 |
| | | | | | | | | |
| Injuries | 3933.7 | 3920.1 | 3989.7 | 4117.8 | 4227.4 | 4091.4 | 4386.8 | 4540.3 |
| IHD | 767.4 | 791.7 | 888.3 | 863.2 | 920.0 | 941.4 | 979.4 | 983.5 |
| Lung cancer | 269.2 | 307.5 | 326.3 | 332.5 | 334.8 | 312.6 | 362.7 | 404.1 |
| Hypertension | 7586.2 | 7268.1 | 7556.6 | 7402.1 | 7286.5 | 7275.6 | 7433.8 | 7494.0 |
| Ulcers | 442.9 | 435.7 | 393.4 | 415.2 | 302.8 | 270.4 | 258.0 | 214.2 |
| Hodgkin's disease | 7.4 | 11.0 | 7.2 | 10.9 | 9.6 | 9.4 | 7.7 | 8.9 |
| Asthma | 321.1 | 259.2 | 229.9 | 203.7 | 148.0 | 115.3 | 97.6 | 74.7 |
| Gallbladder disease | 35.8 | 29.7 | 35.4 | 26.5 | 24.9 | 26.7 | 28.9 | 27.2 |
| Tuberculosis | 1478.3 | 1373.9 | 1264.1 | 1196.2 | 1019.8 | 924.6 | 845.4 | 762.0 |
| Hernia | 15.2 | 11.2 | 12.0 | 6.8 | 9.3 | 7.6 | 9.2 | 6.8 |
| Appendicitis | 21.2 | 10.3 | 9.3 | 13.8 | 10.2 | 13.1 | 5.4 | 8.1 |

| Cause | 1979 | 1980 | 1981 | 1982 | 1983 | 1984 | 1985 | 1986 |
|---|---|---|---|---|---|---|---|---|
| All causes | 19517.6 | 19215.7 | 18943.5 | 18253.6 | 18110.8 | 17304.2 | 16867.1 | 16693.6 |
| | | | | | | | | |
| Injuries | 4711.6 | 4651.6 | 4762.3 | 4497.8 | 4385.4 | 4391.0 | 4220.2 | 4385.5 |
| IHD | 1008.2 | 1260.1 | 1048.5 | 1170.9 | 1265.9 | 1118.8 | 1033.8 | 1306.5 |
| Lung cancer | 384.3 | 424.2 | 443.0 | 455.8 | 463.8 | 474.1 | 492.8 | 485.4 |
| Hypertension | 7144.5 | 6857.5 | 6824.4 | 6706.8 | 6804.8 | 6293.9 | 5922.5 | 5730.4 |
| Ulcers | 202.8 | 187.2 | 189.8 | 167.2 | 173.2 | 171.5 | 136.1 | 117.3 |
| Hodgkin's disease | 9.0 | 5.8 | 6.0 | 5.2 | 8.3 | 5.8 | 3.6 | 5.8 |
| Asthma | 65.9 | 56.1 | 53.7 | 42.2 | 41.6 | 34.6 | 30.1 | 34.1 |
| Gallbladder disease | 24.7 | 22.9 | 19.2 | 17.3 | 22.2 | 19.7 | 16.0 | 16.8 |
| Tuberculosis | 697.0 | 608.7 | 605.5 | 538.6 | 482.8 | 438.5 | 404.8 | 340.4 |
| Hernia | 6.0 | 3.9 | 3.8 | 3.7 | 3.2 | 4.5 | 4.6 | 4.2 |
| Appendicitis | 5.0 | 9.8 | 8.6 | 4.6 | 7.7 | 6.5 | 3.6 | 3.6 |

Additional file 1: Avoidable mortality (SEYLL rate per 100000 people), males, continued

| Cause | Year 1987 | 1988 | 1989 | 1990 | 1991 | 1992 | 1993 | 1994 |
|---|---|---|---|---|---|---|---|---|
| All causes | 16222.2 | 14401.2 | 15593.7 | 14863.3 | 14102.3 | 13869.9 | 13527.1 | 13502.4 |
| | | | | | | | | |
| Injuries | 4486.8 | 4584.5 | 4643.9 | 4375.3 | 4232.7 | 3976.7 | 3929.2 | 3952.1 |
| IHD | 1434.5 | 988.3 | 1055.2 | 1091.0 | 1115.7 | 1270.8 | 1185.5 | 1083.5 |
| Lung cancer | 480.4 | 427.0 | 481.1 | 447.1 | 448.6 | 458.3 | 433.2 | 466.8 |
| Hypertension | 5029.8 | 3783.1 | 4130.2 | 3815.5 | 3441.2 | 3297.2 | 2984.0 | 2866.7 |
| Ulcers | 96.1 | 73.7 | 101.2 | 91.1 | 76.2 | 70.0 | 76.5 | 63.6 |
| Hodgkin's disease | 2.4 | 2.5 | 2.0 | 3.2 | 3.1 | 1.4 | 2.7 | 1.7 |
| Asthma | 32.4 | 33.3 | 28.7 | 30.9 | 35.8 | 32.1 | 34.9 | 37.9 |
| Gallbladder disease | 18.7 | 13.2 | 13.8 | 10.9 | 14.9 | 15.6 | 13.0 | 16.9 |
| Tuberculosis | 312.2 | 251.2 | 286.8 | 257.5 | 226.9 | 204.8 | 171.4 | 168.5 |
| Hernia | 1.3 | 2.0 | 0.9 | 2.4 | 1.7 | 0.6 | 2.1 | 1.0 |
| Appendicitis | 6.4 | 1.9 | 1.9 | 3.9 | 1.4 | 1.4 | 1.8 | 1.5 |

| Cause | 1995 | 1996 | 1997 | 1998 | 1999 | 2000 | 2001 | 2002 |
|---|---|---|---|---|---|---|---|---|
| All causes | 13589.6 | 13390.7 | 12902.2 | 12594.3 | 12390.1 | 11944.4 | 12878.5 | 12260.9 |
| | | | | | | | | |
| Injuries | 3807.1 | 3714.9 | 3369.2 | 3263.3 | 3378.2 | 2944.0 | 2915.6 | 2689.3 |
| IHD | 1003.5 | 1049.0 | 991.2 | 970.1 | 962.8 | 921.6 | 1144.5 | 1090.1 |
| Lung cancer | 480.6 | 497.3 | 504.6 | 475.0 | 483.3 | 465.0 | 580.2 | 582.6 |
| Hypertension | 2889.4 | 2706.4 | 2480.8 | 2330.7 | 2083.0 | 2125.2 | 2422.8 | 2239.7 |
| Ulcers | 64.6 | 65.8 | 66.5 | 61.4 | 53.0 | 57.3 | 52.7 | 48.0 |
| Hodgkin's disease | 2.3 | 3.4 | 2.1 | 2.7 | 2.8 | 2.9 | 2.3 | 3.6 |
| Asthma | 38.9 | 29.4 | 28.0 | 38.0 | 22.4 | 16.4 | 27.3 | 27.2 |
| Gallbladder disease | 9.4 | 12.0 | 9.7 | 10.8 | 14.3 | 10.4 | 13.3 | 13.4 |
| Tuberculosis | 157.7 | 142.6 | 145.9 | 133.4 | 120.7 | 123.5 | 118.1 | 103.9 |
| Hernia | 1.0 | 1.0 | 1.1 | 0.2 | 1.4 | 0.1 | 1.6 | 0.5 |
| Appendicitis | 0.8 | 0.7 | 2.6 | 1.1 | 1.3 | 0.8 | 0.6 | 2.2 |

Additional file 1: Avoidable mortality (SEYLL rate per 100000 people), males, continued

| Cause | Year 2003 | 2004 | 2005 | 2006 | 2007 | 2008 |
|---|---|---|---|---|---|---|
| All causes | 12023.9 | 12076.0 | 12301.9 | 11858.3 | 11467.3 | 11261.8 |
| | | | | | | |
| Injuries | 2562.8 | 2637.0 | 2769.5 | 2672.3 | 2267.9 | 2194.0 |
| IHD | 1096.2 | 1227.8 | 1090.0 | 1031.8 | 989.9 | 989.8 |
| Lung cancer | 564.2 | 541.5 | 544.8 | 544.6 | 562.5 | 555.7 |
| Hypertension | 2184.1 | 2138.0 | 2155.0 | 2040.5 | 1954.9 | 1920.8 |
| Ulcers | 46.5 | 48.0 | 47.3 | 39.1 | 38.6 | 35.8 |
| Hodgkin's disease | 3.9 | 3.7 | 5.1 | 1.9 | 2.6 | 2.3 |
| Asthma | 25.8 | 24.1 | 32.1 | 26.7 | 24.0 | 19.4 |
| Gallbladder disease | 11.4 | 13.6 | 10.4 | 13.1 | 9.5 | 12.6 |
| Tuberculosis | 105.1 | 73.1 | 66.9 | 54.1 | 54.5 | 39.9 |
| Hernia | 0.5 | 0.6 | 0.6 | 0.1 | 0.9 | 0.2 |
| Appendicitis | 1.3 | 1.5 | 1.3 | 1.2 | 0.5 | 1.6 |
