# Supplementary material for: Temporal trend analysis of avoidable mortality in Taiwan, 1971-2008: overall progress, with areas for further medical or public health investment
Source: BMC Public Health. 2013 Jun 6;13:551. doi: 10.1186/1471-2458-13-551 (PMC3744173; doi:10.1186/1471-2458-13-551)
Supplement: Additional file 4 — Avoidable mortality (SEYLL rate per 100000 people), females. National Death Certificate Registry, 1971-2008. Data include cause of death of all mortality in Taiwan from 1971 to 2008. [file 1471-2458-13-551-S4.pdf]

Additional file 4: Avoidable mortality (SEYLL rate per 100000 people), females.

	Year							
Cause	1971	1972	1973	1974	1975	1976	1977	1978
All causes	24081.1	22531.2	22346.3	21524.1	20657.3	20004.0	19583.2	18503.1
Injuries	1823.6	1819.0	1917.7	1846.7	1886.4	1818.7	1895.0	2083.2
Breast cancer	263.5	323.3	281.8	275.3	278.2	279.7	325.5	301.0
Lung cancer	249.7	207.5	223.3	233.9	251.0	232.7	256.5	289.9
IHD	841.2	793.5	904.5	904.2	1005.7	1063.6	1071.3	1126.6
Hypertension	9774.5	10109.1	10094.1	9965.8	9491.2	9633.7	9606.0	9506.8
Cervical cancer	296.0	304.8	292.2	275.7	295.4	305.5	307.6	319.8
Hodgkin's disease	6.0	9.0	2.9	1.9	7.6	3.8	3.0	1.7
Ulcers	322.1	279.2	299.0	286.5	235.0	187.0	177.0	161.7
Asthma	392.3	412.1	342.0	302.3	212.2	173.6	142.9	106.5
Gallbladder disease	65.4	48.5	52.4	43.4	48.7	48.0	51.6	45.6
Maternal mortality	134.6	108.7	119.8	91.9	67.3	89.0	77.9	61.6
Tuberculosis	1020.8	906.1	810.1	733.0	582.9	545.5	482.8	413.9
Hernia	5.0	0.8	2.5	0.7	5.4	5.5	2.8	4.4
Uterine cancer	390.6	317.3	380.0	363.6	329.5	312.5	353.8	290.9
Appendicitis	24.5	20.8	14.1	18.8	18.3	15.1	11.7	9.5

Additional file 2: Avoidable mortality (SEYLL rate per 100000 people), females, continued

	Year							
Cause	1979	1980	1981	1982	1983	1984	1985	1986
All causes	17876.2	17502.3	17128.2	16244.9	15857.5	14719.3	14336.3	13864.0
Injuries	2116.3	2124.7	2131.6	2055.4	2047.7	1932.7	1917.8	2003.8
Breast cancer	356.1	317.5	320.0	311.5	345.7	336.1	360.8	364.1
Lung cancer	247.3	293.1	299.4	305.9	300.7	277.9	294.0	314.1
IHD	1079.7	1127.6	1086.9	1189.0	1163.6	969.1	933.1	1167.7
Hypertension	8868.2	8559.6	8797.7	8176.7	7883.1	7403.3	6891.2	6364.1
Cervical cancer	309.9	332.9	279.4	310.1	308.5	301.4	337.6	305.8
Hodgkin's disease	4.4	5.6	4.4	1.9	5.7	2.1	1.4	2.2
Ulcers	154.3	130.8	133.6	117.9	129.7	113.3	96.7	76.7
Asthma	86.1	61.4	66.3	42.0	45.6	37.3	37.0	43.6
Gallbladder disease	45.7	35.7	30.4	26.3	27.6	31.4	26.6	19.1
Maternal mortality	44.4	46.9	46.1	43.6	36.7	27.3	18.1	14.4
Tuberculosis	344.6	323.1	300.4	267.8	236.1	204.0	166.1	155.4
Hernia	1.1	2.7	2.8	3.6	2.4	1.4	0.7	1.0
Uterine cancer	268.6	260.7	244.3	256.3	200.8	198.1	195.5	157.0
Appendicitis	10.8	10.5	7.2	9.4	9.3	6.1	2.9	3.5

Additional file 2: Avoidable mortality (SEYLL rate per 100000 people), females, continued

	Year							
Cause	1987	1988	1989	1990	1991	1992	1993	1994
All causes	12977.6	9418.1	11719.4	10815.9	9942.6	9398.0	8907.1	8401.7
Injuries	2007.2	1837.4	2015.7	1899.5	1799.5	1686.0	1693.4	1641.5
Breast cancer	354.4	356.0	414.4	410.9	421.2	410.2	458.2	456.7
Lung cancer	302.9	270.0	284.8	263.9	277.8	257.2	257.2	269.6
IHD	1243.1	608.1	859.7	852.9	790.2	844.0	691.3	588.0
Hypertension	5679.7	3274.9	4339.8	3725.3	3126.1	2762.0	2342.3	2088.4
Cervical cancer	294.3	261.9	289.5	297.8	288.0	294.1	288.4	289.9
Hodgkin's disease	2.0	3.5	2.5	1.6	1.2	1.7	0.6	2.6
Ulcers	58.9	35.8	46.5	51.5	37.5	37.1	31.4	27.8
Asthma	36.0	27.0	32.1	28.1	22.7	24.8	29.1	24.4
Gallbladder disease	17.6	11.9	16.1	16.6	17.2	16.0	14.1	12.4
Maternal mortality	14.1	14.1	19.5	19.6	11.8	10.1	13.0	11.4
Tuberculosis	138.8	93.4	95.5	95.7	84.4	75.5	65.1	47.4
Hernia	1.1	0.7	0.9	0.0	0.8	0.4	0.6	0.0
Uterine cancer	147.2	120.5	93.6	70.9	61.4	67.9	55.0	63.8
Appendicitis	4.1	1.8	1.1	2.1	2.9	1.2	1.7	0.6

Additional file 2: Avoidable mortality (SEYLL rate per 100000 people), females, continued

	Year							
Cause	1995	1996	1997	1998	1999	2000	2001	2002
All causes	8477.2	8356.2	7848.0	7514.6	7607.4	7108.3	7486.9	7158.9
Injuries	1640.0	1507.5	1397.9	1407.8	1724.6	1284.1	1203.7	1145.7
Breast cancer	505.0	527.0	543.2	496.6	511.3	533.6	660.0	609.0
Lung cancer	288.1	309.1	309.7	288.8	265.4	287.6	357.5	372.5
IHD	536.9	526.1	481.7	436.1	436.6	386.0	505.3	486.9
Hypertension	2005.2	1858.6	1585.2	1505.2	1305.0	1274.2	1567.3	1375.5
Cervical cancer	301.7	282.6	276.4	267.3	252.2	243.2	269.4	255.2
Hodgkin's disease	1.2	2.4	2.1	0.8	1.4	1.4	2.0	0.6
Ulcers	23.1	27.0	19.5	18.4	24.0	19.8	27.9	25.5
Asthma	26.0	23.0	20.3	23.5	29.3	18.4	21.7	24.0
Gallbladder disease	11.8	6.5	6.6	6.5	9.6	8.2	13.3	9.6
Maternal mortality	11.0	11.1	14.0	10.5	10.4	10.3	7.6	8.6
Tuberculosis	41.2	58.1	48.8	41.2	39.0	33.9	40.8	30.5
Hernia	0.2	0.7	0.3	0.1	0.1	0.4	0.2	0.0
Uterine cancer	50.1	39.1	31.9	44.5	42.1	38.3	45.2	44.7
Appendicitis	0.2	0.5	0.6	1.9	0.5	2.2	1.8	1.0

Additional file 2: Avoidable mortality (SEYLL rate per 100000 people), females, continued

	Year					
Cause	2003	2004	2005	2006	2007	2008
All causes	7027.6	6810.7	6719.8	6273.0	6144.0	5929.5
Injuries	1123.4	1070.3	1147.5	1076.2	969.5	901.8
Breast cancer	667.4	631.0	640.5	621.3	632.7	659.3
Lung cancer	354.6	345.6	323.1	331.2	346.4	342.5
IHD	458.7	479.4	428.9	435.2	376.1	379.9
Hypertension	1363.2	1226.8	1192.3	1044.9	992.1	966.3
Cervical cancer	236.1	222.5	198.2	176.4	172.2	161.6
Hodgkin's disease	1.5	1.2	2.6	1.2	1.9	0.1
Ulcers	21.9	20.5	15.5	20.5	16.2	11.6
Asthma	17.3	14.3	14.5	12.1	13.9	13.5
Gallbladder disease	7.6	10.9	7.7	8.8	8.9	10.0
Maternal mortality	6.2	4.7	6.5	6.2	5.9	5.8
Tuberculosis	31.5	23.0	17.4	16.7	12.5	12.5
Hernia	0.4	0.2	0.8	0.1	0.1	0.0
Uterine cancer	49.8	52.3	62.2	57.9	52.9	64.9
Appendicitis	1.0	1.5	0.5	0.8	1.2	1.2
